# Novel causative mutations in patients with Nance-Horan syndrome and altered localization of the mutant NHS-A protein isoform

**Published:** 2008-10-20

**Authors:** Shiwani Sharma, Kathryn P. Burdon, Alpana Dave, Robyn V. Jamieson, Yuval Yaron, Frank Billson, Lionel Van Maldergem, Birgit Lorenz, Jozef Gécz, Jamie E. Craig

**Affiliations:** 1 Department of Ophthalmology, Flinders University, Bedford Park, South Australia, Australia; 2 Department of Clinical Genetics, The Children’s Hospital at Westmead, Sydney, Australia; 3 Discipline of Paediatrics and Child Health, Faculty of Medicine, University of Sydney, Sydney, Australia; 4 Prenatal Diagnosis Unit - Genetic Institute, Tel Aviv Sourasky Medical Center, Tel Aviv, Israel; 5 Save Sight Institute, Sydney Eye Hospital, University of Sydney, Sydney, Australia; 6 Centre de Génétique Humaine, Université de Liège, Liège, Belgium; 7 Department of Ophthalmology, Universitaetsklinikum Giessen and Marburg GmbH, Giessen, Germany; 8 Department of Genetic Medicine, Women’s and Children’s Hospital, North Adelaide, SA, Australia; 9 Departments of Paediatrics and Molecular Biosciences, University of Adelaide, Adelaide, SA, Australia

## Abstract

**Purpose:**

Nance-Horan syndrome is typically characterized by severe bilateral congenital cataracts and dental abnormalities. Truncating mutations in the Nance-Horan syndrome (*NHS*) gene cause this X-linked genetic disorder. *NHS* encodes two isoforms, *NHS-A* and *NHS-1A*. The ocular lens expresses *NHS-A*, the epithelial and neuronal cell specific isoform. The NHS-A protein localizes in the lens epithelium at the cellular periphery. The data to date suggest a role for this isoform at cell-cell junctions in epithelial cells. This study aimed to identify the causative mutations in new patients diagnosed with Nance-Horan syndrome and to investigate the effect of mutations on subcellular localization of the NHS-A protein.

**Methods:**

All coding exons of *NHS* were screened for mutations by polymerase chain reaction (PCR) and sequencing. PCR-based mutagenesis was performed to introduce three independent mutations in the *NHS-A* cDNA. Expression and localization of the mutant proteins was determined in mammalian epithelial cells.

**Results:**

Truncating mutations were found in 6 out of 10 unrelated patients from four countries. Each of four patients carried a novel mutation (R248X, P264fs, K1198fs, and I1302fs), and each of the two other patients carried two previously reported mutations (R373X and R879X). No mutation was found in the gene in four patients. Two disease-causing mutations (R134fs and R901X) and an artificial mutation (T1357fs) resulted in premature truncation of the NHS-A protein. All three mutant proteins failed to localize to the cellular periphery in epithelial cells and instead were found in the cytoplasm.

**Conclusions:**

This study brings the total number of mutations identified in *NHS* to 18. The mislocalization of the mutant NHS-A protein, revealed by mutation analysis, is expected to adversely affect cell-cell junctions in epithelial cells such as the lens epithelium, which may explain cataractogenesis in Nance-Horan syndrome patients. Mutation analysis also shed light on the significance of NHS-A regions for its localization and, hence, its function at epithelial cell junctions.

## Introduction

Nance-Horan syndrome (NHS; OMIM 302350) is characterized by bilateral congenital cataracts, dental anomalies, craniofacial abnormalities, and, in some cases, mental retardation and behavioral disturbance. This syndrome is caused by mutations in the Nance-Horan syndrome (*NHS*) gene located on Xp22.13 [1-3]. Additional features associated with this disorder are microcornea and microphthalmia [4-6]. While the phenotype of the syndrome in males is severe and necessitates cataract surgery early in life, females display variable severity of cataract and dental defects [6,7]. Since *NHS* has been identified, 16 families affected with this syndrome have been reported worldwide [3,8-11]. In these families, 14 different mutations in four of the eight exons of *NHS* have been identified. All are either non-sense or frameshift mutations that would lead to premature truncation of the protein.

*NHS* encodes two major isoforms, *NHS-A* and *NHS-1A*, which are transcribed by alternate transcription start site usage in exons 1 and 1A, respectively [12]. Individuals who are carrying the disease-causing mutation, 400delC, in exon 1 of the gene are predicted to affect the *NHS-A* isoform alone and exhibit typical features of the syndrome [3,8]. The *Xcat* mouse, a model for NHS, develops X-linked bilateral congenital cataracts due to absence of the *NHS-A* isoform, which resulted from an insertion mutation in intron 1 of *NHS* [13]. Therefore, disruption of the *NHS-A* isoform seems primarily responsible for the syndrome. This epithelial and neuronal cell-specific isoform is expressed in the human lens, the organ severely affected in NHS, and the encoded protein associates with the peripheral cell membrane in the lens epithelium [12]. It co-localizes and interacts with the tight junction protein, ZO (zonula occludens)-1, in epithelium ex vivo, which suggests a role for this isoform at tight junctions ( [12] and unpublished data).

Herein, we report four novel and two recurrent mutations in *NHS* in six unrelated NHS patients. To gain an insight into the functional effects of various *NHS* mutations, two previously reported disease-causing mutations and an artificial mutation were expressed in epithelial cells, and localization of the mutant proteins was determined. While the wild type NHS-A is associated with the cell membrane, the mutant isoform proteins lost this ability, suggesting impaired function as a consequence of protein mislocalization. The present work reveals the significance of various regions of the NHS-A protein for its localization.

## Methods

### Subject recruitment and mutation analysis

The study adhered to the tenets of the Declaration of Helsinki. DNA was obtained from 10 individuals diagnosed with NHS or possible NHS by the referring clinicians (R.V.J., Y.Y., F.B., L.V.M., and B.L.). Where available, DNA from additional family members was collected. Each coding exon and flanking splice site of *NHS* was amplified by polymerase chain reaction (PCR) using intronic primers. Primer sequences and PCR conditions used for each amplimer are given in Table 1. Due to their large size, exon 1 was amplified as three overlapping fragments, exon 6 as eight overlapping fragments, and exon 8 as two. Amplified PCR fragments were purified with the Wizard^®^ SV Gel and PCR Clean-Up System (Promega Corporation, Sydney, Australia). Cleaned fragments were directly sequenced using BigDye Terminators (Applied Biosystems, Foster City, CA) and electrophoresed on an ABI 3100 DNA sequencer (Applied Biosystems). Chromatograms were compared to each other and the reference sequence (GenBank accession number NM_198270) using Sequencher® Software (GeneCodes Corporation, Ann Arbor, MI).

**Table 1:** Primer sequences for the PCR amplification of *NHS* coding regions.

**Exon**	**Amplimer**	**Forward Primer 5′→3′**	**Reverse Primer 5′→3′**	**Size (bp)**	**Mg^2+^ concentration (mM)**	**Annealing temperature (°C)**
1	1A*	GCGCACCCCTAAATTTCT	CTGCCTGCACAGCCATTG	311	2	60
	1B*	GCTTGGAGGAGACCAGAAAGT	CCTGGAAGAGGCTGCAAG	494	2	62
	1C*	GCTGCTCATGCTGGACCTA	GGTGGGAAGGCGAGAGTAGT	349	1.5	60
2	2	TGCAGTAGTCTGGACTTCCACT	CCCATGGATTTCATTTCAGG	426	1.5	60
3	3	ACTCCCAAGGGGAAAAGAGA	CTGATGTTTCCTCAGCAGCA	257	1.5	60
1b	1b	TCTCTCTCTCTGTATGCACCAAA	TCATGCCCTGTTGACTCAGA	352	1.5	60
4	4	GGGCCTATTTCTGACCTCATT	AGCAGCACAGATTTTGAACA	377	1.5	60
5	5	TCTTTCCTTACTTCCCGTCAAA	CATCTGTACTAGGCGGAGGAA	282	1.5	60
6	6A	CTGCCAGCCCACAGATCTAC	GCTTGGAGCCTCACTGAAAT	501	1.5	60
	6B	TCCCCGGGAAGGTAATAGAG	TGAGGGGCTGTGTTTAGTGA	501	1.5	60
	6C	TGACCACCAGTCATCCAGTG	ACCACAGTCAAAGTGCATGG	505	1.5	60
	6D	CATCAGACAAAGCGGACACT	TGGTGGAATATCCGAAGCAT	503	1.5	60
	6E	AATGGAAAACGCCAATCTTC	AGAGGGTTGCTGTAGTGAGGA	502	1.5	60
	6F	CTTGGCATCTCCATCAAGTG	TGCGGCCTAATCTTACTTGG	498	1.5	60
	6G	CCTTGCAATTACACCAACGA	GAGGGTGCCTTCTGTACCTG	534	1.5	60
	6H	AAAACACGCCAACCAAAAAC	ATTCCAGGAAGTGCCATGAG	501	1.5	60
7	7	CAGTAGCGTGCTGGGTAACTT	GGGCAAAACCTTTGTTGTA	235	1.5	60
8	8A	TTTCATAAAAACGTGAACTGAGTGA	GCAAAGCTCTTCGAGGAAAA	489	1.5	60
	8B	CCAGAGGTCTCCTGGTCTCA	GTAAGGGTTTTGGCCTTTGC	511	1.5	60

Exons 1, 6, and 8 were amplified with multiple overlapping amplimers. The expected product size is given along with the required final concentration of Mg^2+^ and the optimal annealing temperature. An asterisk indicates that it required the addition of Q solution (Qiagen).

### Mutagenesis and recombinant DNA cloning

Mutant *NHS-A* cDNA constructs were generated by PCR-based mutagenesis and recombinant DNA cloning. Three mutations, 400delC (exon 1), C2701T (exon 6), and 4071del299bp (exon 6), were incorporated. The position descriptions of the latter two mutations take into consideration the alternatively spliced exon 3a [12]. The wild type *NHS-A* cDNA including the exon 3a sequence was cloned in pCMV-Tag 2A (Stratagene, La Jolla, CA) at EcoRI/SalI sites for use as the parent construct for PCR-based mutagenesis. The sequences of mutagenesis primers are listed in Table 2. The first round of PCR (PCR1 and PCR2) was performed on the parent construct. The primer combinations used for incorporation of each mutation are given in Table 3. PCR1 and PCR2 were performed with the GC-RICH PCR system (Roche Diagnostics Australia Pty Ltd, Castle Hill, NSW Australia) at 95 °C for 3 min, 95 °C for 30 s, at the annealing temperature specified for each primer pair in Table 3 for 30 s, 68 °C for 45 s for 30 cycles, and 68 °C for 7 min. An equimolar mixture of PCR1 and PCR2 products was used as the template for the second round of PCR (PCR3) with the appropriate primer pair for incorporation of each mutation (Table 3). PCR was performed as above except the extension step lasted for 70 s. To replace the corresponding region in the wild type cDNA, the resulting partial mutant cDNAs were independently cloned into the parent construct using restriction enzymes flanking the mutation site (Table 3). The resulting FLAG epitope-tagged wild type and C2701T and 4071del299bp mutant *NHS-A* cDNA were each excised by NotI/PacI digestion and independently cloned in pQCXIN (Clontech Laboratories Inc., Mountain View, CA) at the same sites. The 400delC mutant cDNA (in pCMV-Tag 2A) was digested with EcoRI and EcoRV, and the 1.3 kb digested fragment carrying the mutation was cloned in pEGFP-C1 at EcoRI/SmaI sites to generate a GFP (green fluorescent protein)-fusion construct. The generation of GFP-NHS-A was described elsewhere [12].

**Table 2:** Sequence of primers used for PCR-based mutagenesis.

**Primer**	**Sequence**
NHS-F01	5′-TACCGGAATTCTCCTTTCGCCAAGCGGATCGTGGAG
NHSA400R	5′-GCTGCCGGAGGGTACCGCCAGAGCGGCGTTGCTGA
NHSA400F	5′-ACGCCGCTCTGGCGGTACCCTCCGGCAGCTCTCGGACGT
NHSA1350R	5′-CAGCAATCAGAATATCCTCGGTTTGGCACTCAGAG
NHSA2220F	5′-TTTTAGTCCTGAGCGTCCCAAGGCAGACAG
NHSA2701R	5′-GGAAGATTGGCGTTTTGAATTCAAGAAGGCGTGTTGGCGA
NHSA2701F	5′-GCCAACACGCCTTCTTGAATTCAAAACGCCAATCTTCCCACCA
NHSA3050R	5′-AGCCACTTGATGGAGATGCCAAGCCAGCCA
NHSA4340F	5′-GCAGTCATTCACAGATCCAAGAGGAAAGTACTTG
NHSA4451R	5′-ACATTACTGCTGGGTGAAGAGATCTAACTGGCGCTGCTGCTGCTA
NHSA4451F	5′-CAGCAGCAGCGCCAGTTAGATCTCTTCACCCAGCAGTAATGTGAC
Tag2A810R	5′-TACGACTCACTATAGGGCGAATTGGGTACAC

**Table 3:** The NHS-A mutations generated by PCR-based mutagenesis.

**Mutation**	**Protein length in amino acids (M wt. in kDa)**	**Primer pairs for PCR (annealing temperature °C)**			**Restriction sites for cloning**
		**PCR1**	**PCR2**	**PCR3**	
400delC R134fs)	195 (21.5)	NHSF01 and NHSA400R (56)	NHSA400F and NHSA1350R (58)	NHSF01 and NHSA1350R (65)	EcoRI/EcoRV
C2701T (R901X)	901 (99)	NHSA2220F and NHSA2701R (50)	NHSA2701F and NHSA3050R (52)	NHSA2220F and NHSA3050R (63)	HindIII/PmeI
4071del299bp T1357fs)	1367 (150)	NHSA4340F and NHSA4451R (56)	NHSA4451F and Tag2A810R (56)	NHSA4340F and Tag2A810R (62)	ScaI/SalI

The predicted size of each mutant protein is indicated. The primer combinations used for each round of PCR and the annealing temperature for each primer set is given. The restriction enzyme sites used for the cloning of each partial mutant cDNA are specified.

### Mammalian cell culture

HEK (human embryonic kidney) 293A and MDCK (Madin-Darby canine kidney) cells were cultured in Dulbecco's modified Eagle's medium (DMEM; GIBCO, Invitrogen Australia Pty Ltd, Victoria, Australia), which was supplemented with 10% fetal bovine serum and penicillin/streptomycin, and maintained in a humidified atmosphere at 37 °C and 5% CO_2_.

For the generation of MDCK cells stably expressing the wild type and mutant proteins, 3×10^5^ cells seeded per well in a six well plate were transfected the following day with FLAG-tagged NHS-A, C2701T, or 4071del299bp constructs (in pQCXIN) using Lipofectamine 2000 (Invitrogen Australia Pty Ltd) according to the manufacturer's protocol. Approximately 48 h after transfection, the cells were seeded at 1:20 dilution and cultured in a selection medium containing 0.5 mg/ml G418 (Sigma-Aldrich Pty Ltd, Castle Hill, NSW Australia). Selection was continued for four weeks by changing the selection medium every third day. The resulting population of stable transfectants was used for further experiments.

### Western blot analysis

3×10^5^ HEK 293A cells were seeded per well in six well plates and transfected the following day with wild type, mutant, or appropriate control plasmid using Lipofectamine 2000. Approximately 48 h post-transfection, the cells were harvested and cellular proteins extracted as described elsewhere [12]. Total soluble proteins were size-fractionated on a polyacrylamide gel by SDS–PAGE, and western blot was prepared as previously described [12]. The blot was probed with 1:500 dilution of mouse monoclonal anti-GFP antibody (Roche Diagnostics Australia Pty Ltd) or 1:1000 dilution of mouse monoclonal anti-FLAG tag antibody (GenScript Corporation, Piscataway, NJ) and 1:1000 dilution of sheep anti-mouse IgG-horseradish peroxidase secondary antibody (Chemicon Australia Pty Ltd, Boronia, Victoria, Australia). Antibody binding was detected with ECL western blotting system (GE Healthcare Australia and New Zealand, Sydney, Australia).

### Protein localization

For localization of GFP-fusions, 3×10^5^ MDCK cells were seeded onto glass coverslips in six well plates. Cells were transfected the following day with GFP-fusion constructs or pEGFP-C1 control (Clontech Laboratories Inc.) using Lipofectamine 2000. Approximately 72 h post-transfection, the cells were fixed in 4% paraformaldehyde/phosphate buffered saline (PBS) and mounted on slides in buffered glycerol. Confocal microscopy was performed on an Olympus AX70 microscope (Olympus America Inc., Centre Valley, PA) attached to a Bio-Rad 1024 MRC scanning confocal system (Carl Zeiss Pty. Ltd., North Ryde, Sydney, Australia) equipped with an Argon Ion and a Helium neon laser using LaserSharp 2000 software. GFP was excited with a 488 nm laser line and detected at 522 nm.

For localization of FLAG-tagged wild type and mutant NHS-A proteins, 3×10^5^ MDCK cells stably expressing the protein were seeded onto glass coverslips in six well plates. After culturing for four days, the cells were fixed in 4% paraformaldehyde/PBS, permeabilized with 0.4% Triton-X100, blocked with 5% donkey serum, hybridized with mouse monoclonal anti-FLAG tag antibody (1:1000; GenScript Corporation) and Alexa Fluor 488-conjugated anti-mouse IgG antibody (1:250; Molecular Probes, Invitrogen Australia Pty Ltd, Victoria, Australia), and mounted on slides in buffered glycerol. Confocal microscopy was performed as explained above.

## Results

DNA samples from 10 male individuals with clinical presentations consistent with Nance-Horan syndrome were assessed for mutations in *NHS*. Table 4 shows the clinical features of each patient and the mutation identified. All male patients presented with congenital cataract, and female carriers had sutural cataract. No mutations were identified in individuals 102, 104, 124, and 134 despite features consistent with NHS. Patient 102 had X-linked congenital cataract. Patient 124 appeared to display the NHS phenotype, but characteristic screw-driver blade shaped incisors were not present. Mutations were identified in the remaining six cases.

**Table 4:** Clinical features of NHS patients tested.

**Patient**	**Ocular features**	**Dental features**	**MR**	**FD**	**Mutation**	**Exon**
101	Congenital Cataract Microcornea Microphthalmia Nystagmus Strabismus	-	No	No	K1198fs	6
102	Congenital Cataract	-	-	-	None	
104	Congenital Cataract	Unusual dentition	Yes	Yes	None	
105	Congenital Cataract	Diastema	Yes	Large ears	R879X	6
120	Congenital Cataract Microphthalmia Glaucoma	Diastema Supernumerary Screwdriver-blade shape	No	Yes	R373X	5
122	Congenital Cataract Microphthalmia Strabismus	Supernumerary Abnormal shape	Develop-mental delay	Long thin face	I1302fs	6
124	Congenital Cataract	Supernumerary maxillary incisors	Yes	Large ears	None	
127	Congenital Cataract Microphthalmia	Diastema	No	Prominent ears	P264fs	3
134	Congenital Cataract Microphthalmia	-	-	-	None	
135	Congenital Cataract Microcornea	Supernumerary maxillary incisors	No	High nasal bridge	R248X	3

The mutation identified in *NHS* is shown at the protein level. Missing information is indicated by “-”. MR=mental retardation, FD=facial dysmorphism, fs=frameshift mutation, K=lysine, R=arginine, I=isoleucine, P=proline, X=stop codon.

**Figure 1:**
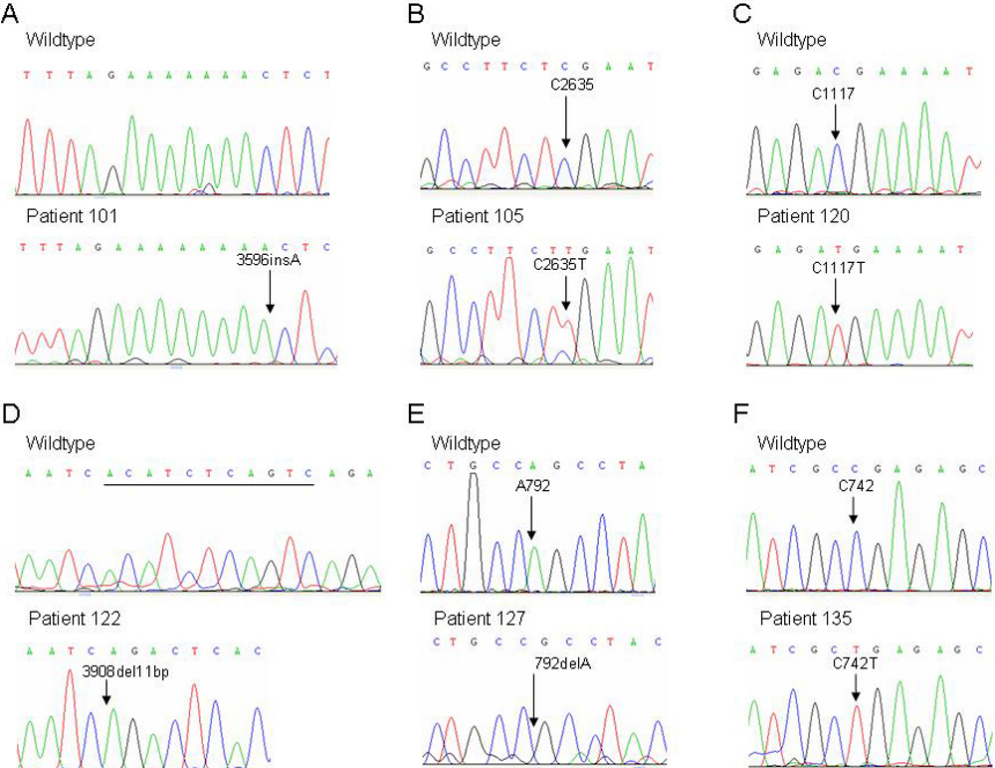
Chromatograms of identified mutations. In each case, the wild type sequence is shown above the mutated sequence for each patient. **A** shows the 3596insA in patient 101; **B** shows the C2635T in patient 105; **C** shows the C1117T in patient 120; **D** shows the 3908del11bp in patient 122; **E** shows the 792delA in patient 127; and **F** shows the C742T in patient 135.

Individual 101, a 19-year-old male from Germany, presented with major ophthalmic features of congenital cataract and secondary glaucoma. He had microcornea and nystagmus at three months of age and presented with strabismus and microphthalmia in childhood. His mother had nuclear cataract with cone-shaped sutural lens opacity. Both the index case and his mother were found to have a previously undescribed single base insertion 3596insA in exon 6, coding for K1198 frameshift (Figure 1A). This mutation results in the incorporation of four aberrant amino acids, terminating at position 1203.

Patient 105 from Belgium had typical features of NHS (congenital cataract, diastema, mental retardation, and large ears). This patient was found to carry the C2635T mutation in exon 6, encoding R879X (Figure 1B). This mutation was previously reported in another family from the Netherlands [10].

Individual 120 from Australia had typical features of NHS. His mother and sister also had cataract and dental anomalies. All three individuals were found to have the R373X mutation, which was previously reported by our group in an unrelated isolated case of NHS from the UK as a de novo mutation [3]. This mutation is designated at the DNA level by C1117T (Figure 1C) and was also reported in a family from the Netherlands [10].

The clinical features of Patient 122, an index case from Israel, have been previously reported [14]. Prenatal diagnosis of cataract in the male fetus of the proband’s sister (the proband’s nephew) led the family to seek genetic diagnosis. The proband displayed many typical features of NHS. Although there was early developmental delay, the patient now has normal intelligence. The novel mutation, an 11 bp deletion at position 3908 (3908delCATCTCAGTCA), in exon 6 causes a frameshift (Figure 1D), resulting in the incorporation of four aberrant residues following I1302 and terminating at position 1307. The proband’s mother and sister are both carriers of the mutation and display a sutural cataract. DNA was also obtained from the affected fetus and shown to carry the same mutation.

Patients 127 and 135 were both referred from Sydney, Australia. Each was found to have a different novel mutation in exon 3 of *NHS*. Patient 127 had bilateral congenital cataract diagnosed soon after birth as well as mild microphthalmia. At 15 months of age, the proband has small widely spaced teeth and prominent ears. His mother and her monozygotic twin sister both had posterior Y-sutural cataracts and had undergone orthodontic procedures for small teeth and irregular crowns. The proband and both women were found to have a single base deletion at position 792 (792delA; Figure 1E). This mutation causes a frameshift following P264. Eighteen aberrant amino acids are predicted to incorporate into the protein that prematurely terminates at position 283. The mutation was not identified in either of the proband’s maternal grandparents, indicating that it arose de novo in his mother and her twin sister.

Patient 135 was described as having both cataract and microcornea. In addition, he had supernumerary maxillary incisors and a high nasal bridge. His mother had bilateral sutural cataracts. A previously undescribed truncating mutation, C742T, was identified in both the proband and his mother (Figure 1F). This mutation resulted in the premature termination of translation at position 248 (R248X). Fifty chromosomes from normal individuals were screened as controls and not found to carry any of the novel mutations identified here in NHS patients.

**Figure 2:**
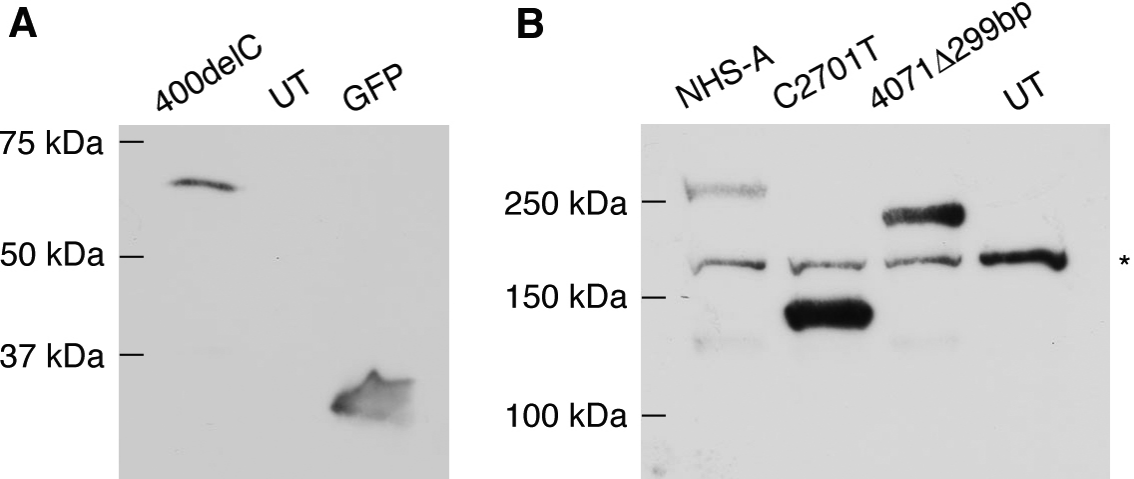
Expression of mutant NHS-A proteins in mammalian cells. **A**: Lysates of HEK 293A cells transiently transfected with GFP-NHS-A400delC and pEGFP-C1 constructs and untransfected cells were analyzed by western blotting with anti-GFP antibody. **B**: Lysates of HEK 293A cells transiently transfected with FLAG-NHS-A, FLAG-NHS-AC2701T and FLAG-NHS-A4071del299bp in pCMV-Tag 2A and untransfected cells were analyzed by western blotting with anti-FLAG tag antibody. A protein band of greater than 150 kDa seen in all the lanes is due to non-specific binding of the anti-FLAG tag antibody (indicated with an asterisk). A very faint protein band of approximately 130 kDa in the NHS-A and 4071Δ299bp lanes is most likely due to protein degradation. The molecular masses of proteins standards are indicated. UT=untransfected cells.

To understand the effect of disease-causing mutations on the NHS-A protein, two previously reported mutations, 400delC (R134fs) in exon 1 and C2635T (R879X) in exon 6 (named C2701T [R901X] in this study because the mutant cDNA included the alternatively spliced exon 3a) [3,8,10], and an artificial mutation, 4071del299bp (T1357fs), also in exon 6 were created in the wild type cDNA by PCR-based mutagenesis. The 400delC mutation was reported in a family with typical features of NHS and is predicted to prematurely truncate only the NHS-A protein isoform. The family with the C2635T mutation displayed most features of the syndrome, and the mutation would abolish the COOH-terminal half of the NHS-A protein. The 4071del299bp mutant was obtained as a cloning artifact while attempting to create the S1484X artificial mutation in exon 8. While cloning the PCR-amplified fragment carrying this mutation to replace the corresponding wild type fragment, a 299 bp deletion was incorporated due to star activity of ScaI, one of the restriction enzymes used for cloning. The resulting 4071del299bp mutation is expected to give rise to a truncated NHS-A protein very similar to that predicted to result from 3908del11bp mutation identified here in patient 122 and was therefore included in the study. The 400delC mutant was fused to GFP at the NH_2_-terminus, and the C2701T and 4071del299bp mutants were fused with a FLAG epitope tag. To confirm that a mutant cDNA expressed the truncated protein, it was transiently expressed in HEK 293A cells and protein expression determined by western blotting. A truncated protein of approximately 65 kDa was detected with an anti-GFP antibody in cells expressing the 400delC mutant (Figure 2A). Similarly, around 140 and 225 kDa truncated proteins were revealed with an anti-FLAG tag antibody in cells respectively expressing the C2701T and 4071del299bp mutants (Figure 2B). These protein sizes are greater than those expected for each of the mutant proteins (Table 3). The wild type NHS-A has a predicted size of 181 kDa but migrates slower than its expected size probably due to post-translational modification (Figure 2B) [12]. The greater than expected size of each mutant protein observed here is therefore not unusual and is also likely to result from post-translational modification.

**Figure 3:**
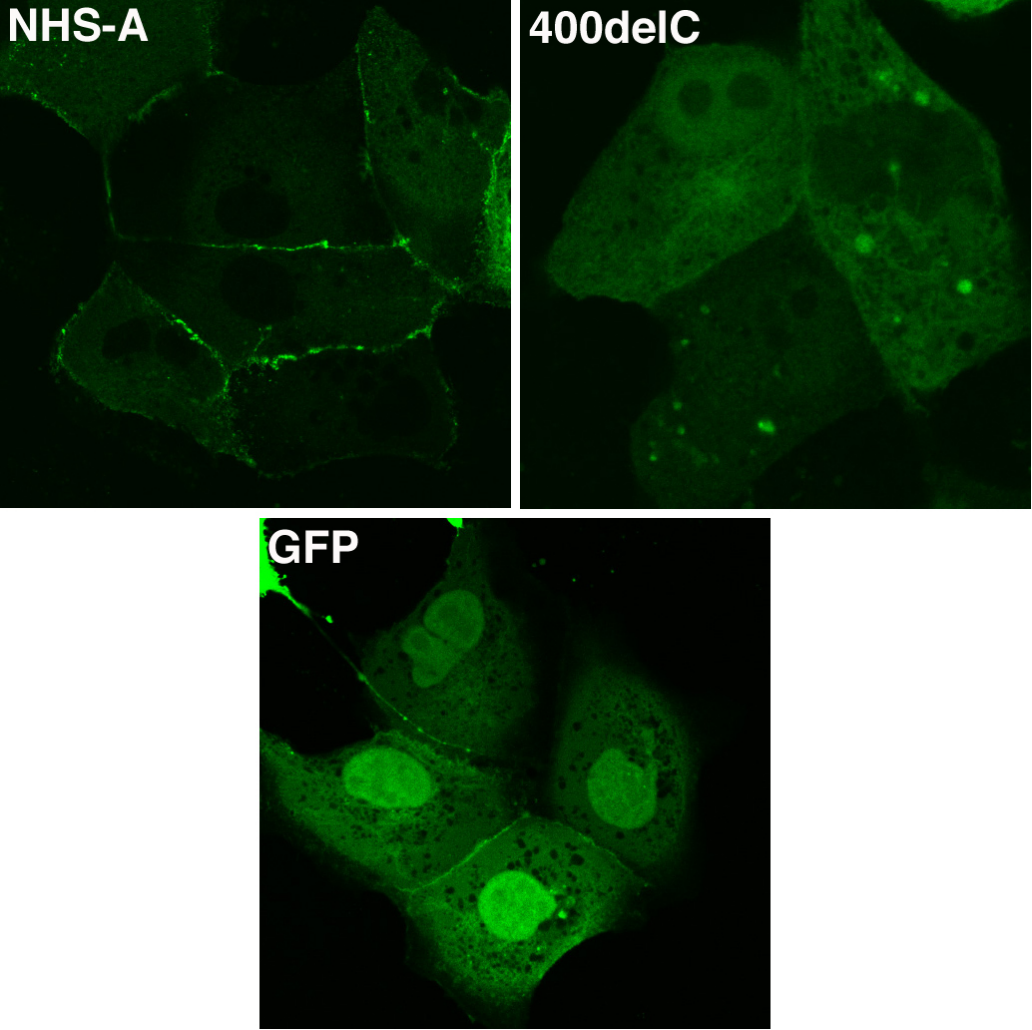
Localization of GFP-NHS-A400delC mutant in MDCK cells. Cells were transfected with GFP-NHS-A and GFP-NHS-A400delC fusion constructs and pEGFP-C1 control. Transiently expressed fusion proteins were visualized by confocal microscopy. GFP-NHS-A wild type protein primarily localized to the cellular periphery whereas GFP-NHS-A400delC mutant protein localized in the cytoplasm and nucleus. Apparent peripheral distribution of GFP is an experimental artifact seen only between some adjoining transfected cells. Images were taken with a 60X objective.

**Figure 4:**
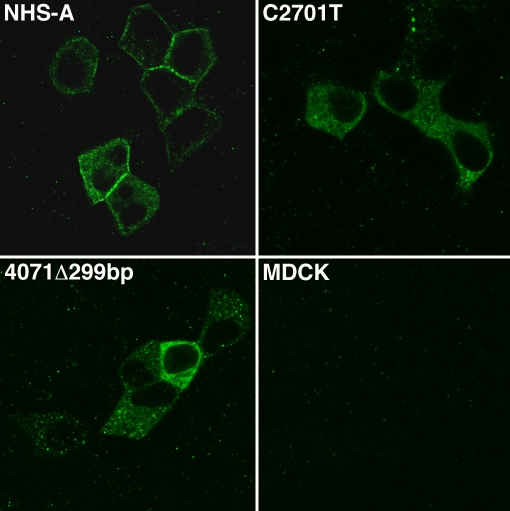
Localization of FLAG-tagged wild type and mutant NHS-A proteins in MDCK cells. MDCK cells stably expressing FLAG-NHS-A, FLAG-NHS-AC2701T, and FLAG-NHS-A4071del299bp proteins and untransfected cells were immunolabeled with an anti-FLAG tag antibody, and labeling was detected by confocal microscopy. Wild type FLAG-NHS-A protein mainly localized to the cellular periphery whereas FLAG-NHS-AC2701T and FLAG-NHS-A4071del299bp mutant proteins localized in the cytoplasm. No non-specific immunoreactivity was observed in untransfected cells. Representative images from four independent experiments are shown. Images were taken with a 60X objective.

To study the subcellular distribution of the mutant proteins, GFP-NHS-A400delC was transiently expressed and FLAG-tagged C2701T and 4071del299bp mutants stably expressed in MDCK epithelial cells. Localization of the mutant proteins was compared with that of the wild type NHS-A protein. Unlike its localization to the peripheral cell membrane in lens epithelium in vivo, the NHS-A protein does not associate with the cell membrane in lens epithelial cells ex vivo because the latter do not form a polarized epithelium in culture [12]. Therefore, localization studies were performed in MDCK cells. As previously reported, transiently expressed wild type GFP-NHS-A primarily localized at the cellular periphery in a punctate fashion in MDCK cells (Figure 3) [12]. Cytoplasmic localization of the protein was seen in some cells. GFP-NHS-A400delC mutant protein mainly distributed in the cytoplasm and nucleus but was not found to associate with the peripheral cell membrane (Figure 3). GFP, expressed as a control, distributed both in the cytoplasm and nucleus. Upon immunolabeling the FLAG-NHS-A stable transfectants with an anti-FLAG tag antibody, intense immunoreactivity was observed at the cell boundary in the majority of cells (Figure 4). In some cells, immunoreactivity was also observed in the cytoplasm. In stable transfectants of FLAG-tagged C2701T and 4071del299bp mutants, FLAG tag immunolabeling was confined to the cytoplasm (Figure 4). No immunoreactivity was observed at the cellular periphery in cells expressing these mutants.

## Discussion

Here, we describe *NHS* mutations in six patients clinically diagnosed with NHS. Four novel mutations (R248X, P264fs, K1198fs, and I1302fs) were identified in four of these patients. The other two (R373X and R879X) represent recurrent mutations and have been previously reported by us and others [3,10]. This is the third report of the truncating R373X mutation, and as such, this region of *NHS* could be a mutation hot spot.

This report brings the total number of known *NHS* mutations to 18, all of which are truncating mutations. All known mutations are located in exons 1, 3, 5, and 6 with no obvious phenotype/genotype correlation. The apparent absence of missense mutations in *NHS* is intriguing. It is possible that missense mutations give rise to a different (perhaps milder) phenotype such as isolated X-linked cataract or X-linked mental retardation, although no reports of this have been presented to date. Ten patients with a clinical diagnosis of NHS were screened for mutations in this study. Mutations were identified in only 60% of patients. Patients like 102 and 134 with only ophthalmic information available and no identified mutation may not represent NHS cases. At least one patient, 124, has all the features of the syndrome but no identified mutation while other patients such as 127 and 135 with superficially similar phenotypic features have a mutation in *NHS*. Thus, a clinical diagnosis is not always significant in predicting a mutation in this gene. It is as yet unknown if patients like 124 represent phenotypic overlap of other syndromes with NHS or if mechanisms other than protein truncating mutations in *NHS* can cause the phenotype. Alternative mechanisms may include mutations in the introns such as that present in the *Xcat* mouse or in the promoter or 5′ and 3′ untranslated regions of *NHS*, which have not been investigated here due to the very large size of the gene and unidentified regulatory elements. Patients negative for mutations in the *NHS* gene in this study were not screened for mutations in other genes.

The two disease-causing mutations, 400delC and C2701T, and the artificial mutation, 4071del299bp, analyzed here, respectively, reside in the NH_2_-terminus, middle, and COOH-terminus of the NHS-A protein. All mutant proteins lost the ability to associate with the cell membrane at the cell boundary, which is exhibited by the wild type NHS-A protein in epithelial cells, and instead localized in the cytoplasm. This aberrant distribution of the mutant proteins may affect the integrity of intercellular junctions in those epithelial cells that express NHS-A such as the anterior lens epithelium. Interestingly, in an attempt to generate MDCK cells stably expressing the FLAG-tagged 400delC mutant upon immunolabeling with the anti-FLAG tag antibody, no positive cells were detected after G418 selection (data not shown). We speculate this may be due to the degradation of the mutant protein. Hence, this protein was transiently expressed as a GFP-fusion for localization studies. Nuclear localization of GFP-NHS-A400delC noted here might be due to overexpression of the fusion protein.

We previously reported the NH_2_-terminal 374 amino acids of NHS-A to be responsible for its peripheral membrane-associated localization in epithelial cells [12]. However, in this study, the R134fs (400delC) mutant protein carrying the first 134 residues localized in the cytoplasm (Figure 3). This mislocalization of the mutant protein may be due to lack of amino acids 135-374. However, the possibility of mislocalization due to the incorporation of aberrant amino acids after the frameshift cannot be excluded. Cytoplasmic localization of the truncated proteins, R901X (C2701T) and T1357fs (4071del299bp), despite the presence of the NH_2_-terminal 374 residues may be due to protein misfolding (Figure 4). Both these truncated proteins lack the COOH-terminal 295 amino acids from position 1358 to position 1652, which may lead to their misfolding and hence mislocalization. Because a GFP-fusion of the NH_2_-terminal 374 residues of NHS-A associates with the cell membrane [12] but the R901X and T1357fs mutant proteins do not, the downstream residues in these proteins may interfere with their folding in the absence of the COOH-terminus. Taken together, these data indicate the significance of three regions in the NHS-A protein for its localization. The NH_2_-terminus is necessary for peripheral membrane associated localization of the protein [12], the middle region causes mis-localization of the protein in the absence of the COOH-terminus perhaps by exerting an inhibitory effect, and the COOH-terminus seems to be important for proper localization of the protein probably by facilitating its folding. Therefore, both the NH_2_- and COOH-termini of the NHS-A protein are important for its peripheral membrane-associated localization in epithelial cells and its function at cell-cell junctions. In view of these findings, the disease-causing mutations in *NHS* identified in NHS patients in the present and previous studies would cause mislocalization of the mutant NHS-A protein in epithelial cells that express this isoform such as the lens epithelium. Mislocalization of the protein may in turn impact epithelial cell junctions in the developing lens and other organs affected in this genetic disorder.
